# The Brazilian Caatinga Biome as a Hotspot for the Isolation of Antibiotic-Producing *Actinomycetota*

**DOI:** 10.3390/life15101494

**Published:** 2025-09-23

**Authors:** Sayoane Pessoa Fernandes, Luana Layse Câmara de Almeida, Adrielly Silva Albuquerque de Andrade, Lucas Silva Abreu, Yuri Mangueira Nascimento, Thalisson Amorim de Souza, Evandro Ferreira da Silva, Fabiana Caroline Zempulski Volpato, Afonso Luis Barth, Josean Fechine Tavares, Demetrius Antonio Machado de Araújo, Valnês da Silva Rodrigues-Junior, Samuel Paulo Cibulski

**Affiliations:** 1 Programa de Pós-Graduação em Ciências Farmacêuticas, Departamento de Farmácia, Universidade Estadual da Paraíba (UEPB), Campina Grande 58429-500, PB, Brazil; pessoasayoane@gmail.com (S.P.F.); lu.laysec@gmail.com (L.L.C.d.A.); 2 Laboratório de Biotecnologia Celular e Molecular, Centro de Biotecnologia, Universidade Federal da Paraíba (UFPB), João Pessoa 58051-900, PB, Brazil; adriellysaa@gmail.com (A.S.A.d.A.); demetrius@cbiotec.ufpb.br (D.A.M.d.A.); 3 Departamento de Química Orgânica, Instituto de Química, Universidade Federal Fluminense (UFF), Niterói 24020-007, RJ, Brazil; lucas.abreu@ltf.ufpb.br; 4 Laboratório Multiusuário de Caracterização e Análise (LMCA), Instituto de Pesquisa em Fármacos e Medicamentos, Universidade Federal da Paraíba (UFPB), João Pessoa 58051-900, PB, Brazil; yurimangueira@ltf.ufpb.br (Y.M.N.); thalisson.amorim@ltf.ufpb.br (T.A.d.S.); evandro@ltf.ufpb.br (E.F.d.S.); josean@ltf.ufpb.br (J.F.T.); 5 Departamento de Biociências, Universidade Federal do Paraná (UFPR), Setor Palotina, Palotina 85950-000, PR, Brazil; fabiana_volpato@yahoo.com.br; 6 Laboratório de Pesquisa Em Resistência Bacteriana (LABRESIS), Hospital de Clínicas de Porto Alegre, Porto Alegre 90035-007, RS, Brazil; albarth@hcpa.edu.br; 7 Programa de Pós-Graduação em Produtos Naturais e Sintéticos Bioativos, Universidade Federal da Paraíba (UFPB), João Pessoa 58051-900, PB, Brazil; 8 FACISA—Faculdade de Ciências da Saúde do Trairi, Universidade Federal do Rio Grande do Norte (UFRN), Santa Cruz 59200-000, RN, Brazil

**Keywords:** Brazilian Caatinga biome, antibiotic-producing *Actinomycetes*, anti-tubercular agents, molecular networking

## Abstract

Antimicrobial resistance represents a critical global health challenge, intensifying the urgency of discovering novel antibiotics. *Actinomycetota* species, the most prolific source of clinical antibiotics, remain underexplored in unique ecosystems. In this study, we isolated 340 *Actinomycetota* strains from soils of the Brazilian semiarid Caatinga biome. Screening revealed that 122 isolates (35.9%) exhibited antimicrobial activity against clinically relevant pathogens (*Escherichia coli*, *Pseudomonas aeruginosa*, *Staphylococcus aureus*, and *Candida albicans*). Notably, 19 isolates showed activity against *Mycobacterium tuberculosis* H37Ra. MALDI-TOF MS analysis successfully provided genus-level identification for a subset of isolates, with approximately 32% assigned to the *Streptomyces* genus. However, the limited resolution of the database for the majority of the strains indicates high phylogenetic diversity and suggests the presence of potentially novel species. Metabolomic profiling via LC-MS/MS and GNPS molecular networking suggested the production of known antibiotics such as actinomycins, cyclomarins and anthracyclines and unveiled distinct molecular families putatively assigned to undescribed metabolites. Our work establishes the Caatinga biome as a valuable reservoir of bioactive *Actinomycetota*, encoding both known and potentially novel antimicrobial compounds. These results underscore the potential of underexplored and extreme environments in the quest to overcome antibiotic resistance.

## 1. Introduction

Antimicrobial resistance (AMR) is recognized by the World Health Organization (WHO) as one of the top 10 global health threats. This silent pandemic of drug-resistant infections disproportionately affects low- and middle-income countries and vulnerable populations, including children and the elderly [1]. If unaddressed, AMR could compromise the treatment of common infections and make routine medical procedures increasingly risky. Research on antibiotics is therefore critical to counteract resistance and ensure preparedness for emerging infectious challenges.

Combating AMR demands coordinated interdisciplinary efforts. Microbiologists, molecular biologists, chemists, clinicians, epidemiologists, and economists, alongside governments and international organizations, must collaborate to discover, responsibly use, and preserve antibiotics. Such integrated action is essential to slow the rise of resistance and safeguard the continued efficacy of these life-saving drugs [2].

In this context, the *Actinomycetota* represent a critical resource, as this phylum of Gram-positive bacteria produces a milieu of bioactive metabolites, including antibiotics (e.g., streptomycin, rifamycins, and erythromycin), antiproliferative drugs (e.g., doxorubicin, mitomycin, and bleomycin), immunosuppressive drugs (e.g., rapamycin and tacrolimus), and other important medical molecules [3]. More than 25,000 bioactive secondary metabolites from microorganisms have been identified, and about 70% of these are produced by *Actinomycetota* species, including more than a hundred antibiotics used in human and animal therapy and agriculture [4]. However, the use of general and classical approaches to discover and develop new drugs from these microorganisms is becoming increasingly complex and demanding. To circumvent the challenges of drug development from *Actinomycetota*, new concepts based on the triad “genomics, chemical dereplication and new habitats” have been described [5]. In other words, new habitats harbor new species, which should contain new gene clusters synthesizing novel secondary metabolites. The alliance of simple, easy, and cheap classical microbiological methods with modern genomic and metabolomics techniques that focus on *Actinomycetota* species from new habitats, such as oceans, extreme environments including arid regions, plants, and feces of animals and lichens, could be a perfect marriage, offering hope for the development of novel drugs to combat old and new enemies.

According to the United Nations Environment Programme (UNEP), Brazil is recognized as the most biologically diverse country on Earth, hosting an estimated 15–20% of all known animal and plant species. While the country’s macrodiversity has been increasingly documented, its microbial diversity remains largely unexplored [6]. Microorganisms represent a largely untapped reservoir of bioactive compounds, offering immense potential for the discovery of novel drugs and other molecules of medical and biotechnological significance. Exploring this hidden microbial diversity is therefore critical to harnessing Brazil’s full biotechnological potential. Therefore, considering (i) the urgent need for novel, potent, and safe antibiotic therapies in the face of the rising burden of multidrug-resistant infections, (ii) the fact that many of the most effective antibiotics were originally discovered in *Actinomycetota*, and (iii) the largely unexplored and highly diverse Brazilian habitats that may harbor antibiotic-producing *Actinomycetota*, we applied classical microbiological methods to isolate such microorganisms from an “extreme” Brazilian semiarid biome, the Caatinga.

Thus, the aim of this study was to isolate and characterize *Actinomycetota* from the Caatinga biome, screen their antimicrobial and anti-tubercular potential, and employ metabolomic profiling to dereplicate known compounds and prioritize isolates for the discovery of potentially novel metabolites. By combining the strategy of targeting microorganisms from underexplored environments with modern dereplication approaches, this work aims to identify promising candidates for future development into new therapeutic agents against resistant infections.

## 2. Material and Methods

### 2.1. Collection of Soil Samples and Isolation of Actinomycetota

Six soil samples were collected aseptically from six different sites within the Caatinga biome in Paraíba State, Brazil (coordinates: #1: 7.31893° S, 36.39578° W; #2: 7.39248° S, 36.52796° W; #3: 7.40133° S, 36.52487° W; #4: 7.43368° S, 36.48676° W; #5: 7.49937° S, 36.34497° W; #6: 7.47623° S, 36.28489° W). Details from study area are described in Supplementary Figure S1A. Samples were retrieved from the upper layer of soil (~15 cm) using sterile plastic bags and immediately transported to the laboratory for further isolation steps. The soil collection and isolation procedures were performed in October 2018, in the dry season of Caatinga (Supplementary Figure S1B).

*Actinomycetota* isolation was performed using standard microbiological techniques. Briefly, 10 g of each soil sample was diluted with 90 mL of saline solution and homogenized in rotary shaker at 230 rpm for 1 h. Then, a 10 mL aliquot was heated to 65 °C during 5 min and serial dilutions up to 10^−4^ were carried out in saline solution. An amount of 100 μL from 10^−2^, 10^−3^, and 10^−4^ dilutions was spread on soil–agar (pH 7.0) and tap water–agar (pH 10.0) (HiMedia, Mumbai, India) and incubated at 30 °C for 7 days. Soil extracts were prepared from soil rich in organic matter, free of roots and debris. Briefly, 400 g of soil was mixed with 1 L of water, autoclaved at 121 °C for 60 min, cooled, filtered, and autoclaved again at 121 °C for 20 min. The sterile extract was stored at room temperature. Soil–agar was prepared by dissolving 15 g of bacteriological agar (HiMedia, Mumbai, India) in 1 L of the sterile soil extract and autoclaving at 121 °C for 15 min.

Colonies with a tough or powdery texture, dry or folded appearance and branching filaments with or without aerial pseudo mycelia were sub-cultured. A re-purification procedure was performed using the streak plate method and then the isolate was stored in 40% glycerol at −80 °C. Standard Gram staining was applied to verify the filamentous nature of the isolates. The microorganisms were registered in the National System for the Management of Genetic Heritage and Associated Traditional Knowledge (SisGen) under number A9E11C6.

### 2.2. Antimicrobial Activity Screening

The antimicrobial activity of the *Actinomycetota* isolates was evaluated against *Staphylococcus aureus* ATCC 25923, *Escherichia coli* ATCC 25922, *Pseudomonas aeruginosa* ATCC 27853, and *Candida albicans* ATCC 10231. Two complementary methods were employed.

For *E. coli*, *P. aeruginosa*, and *C. albicans*, the cross-streaking antibiosis assay was performed as described by Waksman [7]. Briefly, each *Actinomycetota* isolate was streaked as a single line onto M9 agar plates and incubated at 28 °C for 14 days. After incubation, indicator strains were streaked perpendicularly to the *Actinomycetota* growth line. Plates were incubated under optimal conditions (37 °C for bacteria, 30 °C for *C. albicans*) and inhibition of growth at the intersection was recorded. The M9 minimal salt solution was prepared as follows: 64 g/L Na_2_HPO_4_·7H_2_O, 15 g/L KH_2_PO_4_, 2.5 g/L NaCl, 5.0 g/L NH_4_Cl and 1.5% of bacteriological agar (HiMedia, India). After autoclaving, the following filter-sterilized solutions were added to the specified final concentrations: 2 mL/L of 1 M MgSO_4_, 0.1 mL/L of 1 M CaCl_2_, and 20 mL/L of 20% glucose (as a carbon source). The pH was adjusted to 7.0 prior to the addition of agar (15 g/L).

For *S. aureus*, the agar block/plug assay was performed on Mueller–Hinton agar, a nutrient-rich medium required due to its higher growth demands compared to the actinomycete indicator strains grown on M9 agar [8]. *Actinomycetota* isolates were cultured on M9 agar at 28 °C for 14 days, and 6 mm plugs of agar containing *Actinomycetota* growth were aseptically removed and placed on Müeller–Hinton agar plates previously seeded with *S. aureus*. Plates were incubated at 37 °C for 24 h, and antimicrobial activity was determined by measuring zones of inhibition around the plugs. All assays were performed in triplicate. Isolates were considered active when consistent inhibition was observed in at least two independent assays.

### 2.3. Anti-Tubercular Potential of Actinomycetota Extracts

Each *Actinomycetota* isolate was subcultured in soil–agar until sporulation (~7 days), and then spores were inoculated in deep-well plates containing 2 mL of M9 media and then incubated on rotary shaker incubator (200 rpm) at 37 °C for fourteen days. After, the culture was centrifuged at 3000× *g* for 1 h. The cell-free broth obtained was extracted three times with ethyl acetate (EtOAc, Dinâmica, Indaiatuba, SP, Brazil). The organic phase was concentrated to dryness, dissolved in 100 µL of dimethyl sulfoxide (DMSO) and used for anti-tubercular screening as further described.

Anti-mycobacterial activity was assessed using *Mycobacterium tuberculosis* H37Ra and *Mycobacterium smegmatis* mc^2^155 strains, determined using the resazurin reduction microplate assay (REMA) as a growth indicator, as previously reported [9]. For safety reasons, the anti-tubercular assays were performed using the attenuated *M. tuberculosis* H37Ra strain; while suitable for initial screening, this strain may not fully reflect the clinical behavior of virulent strains. Briefly, mycobacterial suspensions were cultivated and diluted in Middlebrook 7H9 medium (BD Difco, USA) plus ADC enrichment (albumin, dextrose, catalase; BD Difco, Franklin Lakes, NJ, USA) at an optical density (OD_595_ nm) of 0.001 for *M. smegmatis* and 0.006 for *M. tuberculosis*, and 200 μL was added to each well. Further, 5 μL of DMSO-diluted *Actinomycetota* extract was added to wells. Following incubation at 37 °C for 24 h for *M. smegmatis*, or 7 days for *M. tuberculosis*, 30 μL of a sterile resazurin solution (0.02%) was added to the plates and the results were evaluated after 24 h for *M. smegmatis*, or 48 h for *M. tuberculosis* [10]. Growth inhibition activity was considered when the *Actinomycetota* extract added prevented a color change from blue (resazurin) to pink (resorufin). The minimum inhibitory concentration (MIC) of the control drugs isoniazid and rifampicin (both purchased from Sigma-Aldrich, St. Louis, MO, USA) were determined in each independent test. Three independent experiments were performed.

### 2.4. Microbial Identification Using Matrix-Assisted Laser Desorption Ionization–Time of Flight Mass Spectrometry (MALDI-TOF MS)

For MALDI-TOF MS identification, microbial isolates (Table 1) were grown in Luria–Bertani (LB) broth for 24–48 h under agitation. The biomass was collected by centrifugation, transferred onto a MALDI target plate, and overlaid with 1 μL of sinapinic acid matrix solution. Mass spectra acquisition was performed using a Microflex LT mass spectrometer (Bruker Daltonics, Bremen, Germany) and the Flex Control software (version 3.4) with default parameter settings. Evaluation of the mass spectra was carried out using the Flex Analysis and MALDI Biotyper 3.1 software and library (version 3.1.66). The standard MALDI Biotyper^®^ interpretative criteria were applied as follows: unreliable identification (score 0.000–1.699); probable genus identification (score 1.700–1.999); secure genus and probable species identification (score 2.000–2.299); and highly probable species identification (score 2.300–3.000).

### 2.5. LC-MS/MS Analyses and Molecular Networking

**Figure 1:**
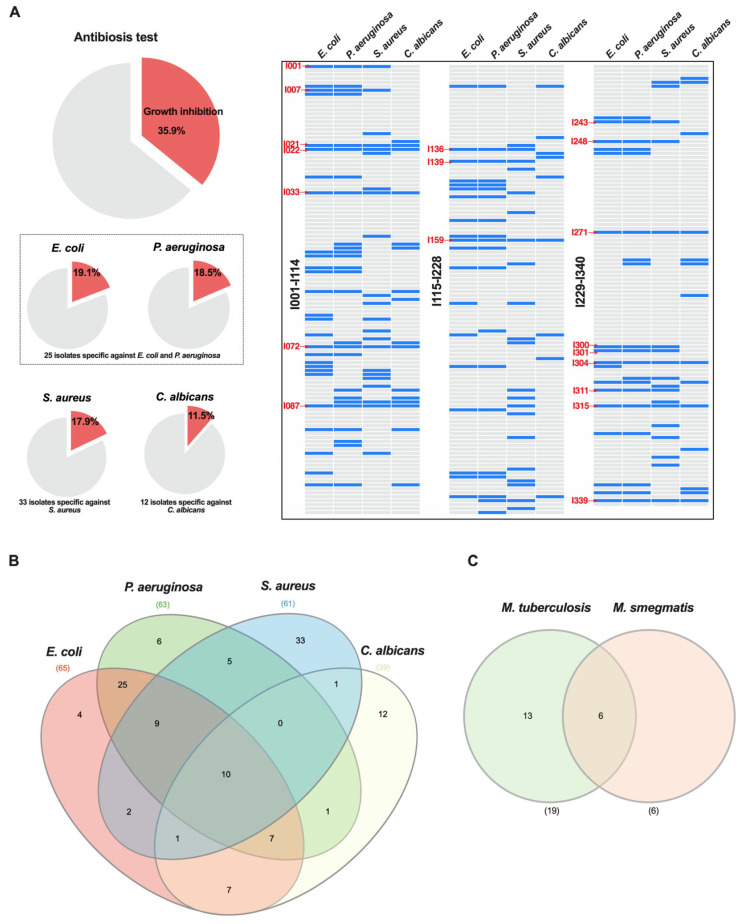
**Antimicrobial activity and of *Actinomycetota* isolates from Caatinga soils.** (**A**) Antimicrobial activity of 340 *Actinomycetota* isolates against *E. coli*, *P. aeruginosa*, *S. aureus*, and *C. albicans*. Pie charts indicate the percentage of isolates exhibiting inhibitory activity for each indicator microorganism. Heatmaps show the activity profile of the isolates (I001-340), with blue bars representing growth inhibition. Red labels indicate isolates with broad activity against both Gram-positive and -negative pathogens. (**B**) Venn diagram depicting the overlap of *Actinomycetota* isolates active against the four tested microorganisms. Numbers indicate the quantity of isolates inhibiting one or more pathogens. (**C**) Venn diagram showing isolates with antimycobacterial activity.

Nineteen isolates of *Actinomycetota* able to exhibit a broad inhibition spectrum (highlighted in Figure 1A) plus isolates that inhibited *M. tuberculosis* were selected to LC-MS/MS analysis. *Actinomycetota* isolates were initially cultured on soil–agar until sporulation (~7 days). Spores were then inoculated into 125 mL Erlenmeyer flasks containing 50 mL of M9 medium and incubated on a rotary shaker at 200 rpm and 37 °C for 14 days. Following incubation, cultures were centrifuged at 3000× *g* for 20 min. The resulting cell-free supernatant was extracted three times with EtOAc, and the combined organic phases were concentrated to dryness under reduced pressure.

A Shimadzu^®^ (Kyoto, Japan) High-Performance Liquid Chromatography (HPLC) system coupled to an AmazonX (Bruker Daltonics, Billerica, MA, USA) equipped with an electrospray ionization (ESI) source was used for ESI-MS^n^ analysis. The LC system comprised an LC-20AD solvent pump (flow rate 600 µL/min), DGU-20A5 online degasser, CBM-20A system controller, and an SPD-M20A diode array detector (190–800 nm) (Shimadzu^®^, Kyoto, Japan). Separation was performed on a Kromasil C18 column (5 µm, 100 Å, 250 × 4.6 mm, Kromasil, Uppsala, Sweden). Injections of 20 µL (1 mg/mL in acetonitrile) were performed using an autosampler (SIL-20A). The mobile phase consisted of 0.1% formic acid in water (solvent A) and acetonitrile (solvent B), using a linear gradient from 5–100% B over 60 min.

ESI-MS^n^ parameters were as follows: capillary voltage 4.5 kV, positive ion mode, final plate offset 500 V, nebulizer 4.0 bar, dry nitrogen gas at 8 mL/min, and a temperature of 200 °C. Collision-induced dissociation (CID) fragmentation was performed in auto MS/MS mode using advanced resolution for both MS and MS/MS. Spectra were recorded every 2 s over *m*/*z* 50–1500. MS data were processed using Bruker Data Analysis software v4.2.

LC-MS/MS data were converted to mzXML using MSConvert and uploaded to the Global Natural Product Social (GNPS) molecular networking platform [11]. Molecular networking was generated with precursor and fragment ion mass tolerances of 0.8 and 0.2 Da, respectively. Advanced network parameters included minimum pair cosine 0.7, TopK 10, maximum connected component size 100, maximum matched fragment ions 4, and minimum cluster size 2. For library searches, a minimum of 6 matching peaks with a score threshold ≥ 0.7 was used. All other parameters were kept at default values. Networks were visualized in Cytoscape v3.6.1.

To enhance chemical structural information within the molecular network, information from in silico structure annotations from GNPS Library Search were incorporated into the network using the GNPS MolNetEnhancer workflow [12].

## 3. Results and Discussion

### 3.1. Recovery of Actinomycetota from the Caatinga Biome

Using simple, inexpensive, and classical methodologies for microbial isolation, a total of 340 *Actinomycetota*-like colonies were recovered from soil samples collected in the Brazilian semiarid region (Caatinga). On average, 56 isolates were obtained per soil sample (ranging from 43 to 78 colonies). Regarding the isolation media, 66.7% of the *Actinomycetota* were recovered from tap water–agar plates (pH 10.0), while the remaining isolates grew on soil–agar plates (Supplementary Figure S2). Most isolates exhibited typical *Actinomycetota* features, such as production of pseudo-hyphae, dry and powdery colonies, and pigment production, the latter often diffusing into the culture medium (Supplementary Figure S3A,B). Tap water–agar showed high selectivity, as >95% of the colonies displayed *Actinomycetota*-like morphology. Conversely, soil–agar plates also supported the growth of *Bacillus*-like colonies (Gram-positive rods) and *Paenibacillus vortex*-like colonies, indicating lower selectivity.

The high recovery of *Actinomycetota* under these conditions highlights the Caatinga biome as a potential reservoir of microorganisms well adapted to environmental stress. The selective performance of the tap water–agar (pH 10.0) is consistent with its oligotrophic composition, which limits the growth of fastidious bacteria and mimics the nutrient-poor conditions of dry soils. In contrast, soil–agar, a richer medium, allowed the proliferation of other spore-forming bacteria. These results reinforce the importance of tailoring cultivation strategies when targeting *Actinomycetota* in complex microbiomes.

The sampled region is located within the core of the Brazilian Caatinga, a seasonally dry tropical forest and an exclusive biome that represents one of the world’s most extensive and biodiverse semiarid ecosystems [13]. It is characterized by a hot semiarid climate (BSh, Köppen classification), with a mean annual precipitation of only 464.8 mm and average temperatures exceeding 26.5 °C (Supplementary Figure S1A). Soils were collected during an exceptionally dry period (October 2018), after approximately five months without rainfall (Supplementary Figure S1B). This ecosystem exhibits remarkable climatic seasonality, with a short, irregular rainy season followed by a prolonged and intense dry period that can last up to 11 months, imposing strong abiotic filters on the soil microbial community. Such conditions are known to favor drought-tolerant bacterial groups, particularly *Actinomycetota*, which are routinely enriched under drought due to traits such as spore formation and strong cell walls [13] and *Bacillus* spp., which are frequently selected in arid ecosystems and known to enhance plant drought resistance via hormonal and metabolic mechanisms [14].

While other underexplored and extreme biomes, such as deserts (e.g., Atacama), mangroves, and caves, are recognized reservoirs of microbial novelty, the Caatinga presents a unique combination of abiotic pressures. Unlike hyper-arid deserts, the Caatinga is a seasonally dry tropical forest characterized by intense, predictable interannual drought cycles punctuated by irregular, heavy rainfall events ecosystems [13]. This creates a powerful cycle of dehydration and rehydration stress. Furthermore, it experiences consistently high solar irradiation and elevated soil temperatures year-round. While mangroves exert strong osmotic pressure, the Caatinga’s stress is primarily thermohydric. We hypothesize that this specific combination of extreme UV exposure, cyclic drought, and high temperatures selects for a microbial community with specialized adaptations distinct from those found in other well-studied extreme environments. This is reflected in our initial finding that a significant portion of our isolates were not identifiable via MALDI-TOF MS, suggesting genetic and metabolic divergence (discussed in detail in a dedicated section).

From an ecological perspective, the abundance of pigmented and sporulating *Actinomycetota* suggests adaptive strategies against UV stress and desiccation. Similar patterns have been observed in polyextremophilic *Actinomycetota* isolated from high-altitude Andean lakes, where many isolates displayed resistance to multiple environmental stress factors, including intense UV-B radiation, highlighting pigmentation and spore formation as critical survival mechanisms in harsh, sun-exposed habitats [15]. Pigment production and powdery colony morphology are not only survival traits but are also often linked to secondary metabolite biosynthesis. Similar findings have been reported in desert environments, where *Actinomycetota* contribute to soil resilience and are recognized as prolific producers of bioactive compounds. Indeed, deserts are increasingly acknowledged as underexplored sources of diverse *Actinomycetota* taxa with high biotechnological potential, having already yielded over 50 novel compounds with antibacterial, antiviral, antitumor, anti-inflammatory, and other bioactivities [16]. More broadly, *Actinomycetota* isolated from extreme habitats such as deserts, caves, and mangroves have consistently been demonstrated as prolific producers of antibiotics, immunosuppressive agents, enzymes, and other secondary metabolites [17]. In particular, the Atacama Desert has already produced a notable diversity of natural products derived from its resident *Actinomycetota* communities [18,19,20]. Thus, the recovery of a large and morphologically diverse set of isolates from Caatinga soils indicates that this biome may represent a promising, yet underexplored, source of metabolically versatile *Actinomycetota* with potential for biotechnological applications.

### 3.2. Antimicrobial Activity Spectrum of Actinomycetota Isolates from Caatinga Biome

Among the 340 *Actinomycetota* isolates screened, 35.9% (122/340) exhibited antimicrobial activity against at least one medically relevant microorganism (Figure 1A,B). Approximately 19% of isolates produced metabolites capable of inhibiting Gram-negative bacteria (*E. coli*, 65 isolates; *P. aeruginosa*, 63 isolates). Among them, 25 isolates showed activity exclusively against Gram-negative bacteria, without inhibiting *S. aureus* or *C. albicans*, suggesting the production of selective bioactive compounds. For *S. aureus*, 61 isolates (17.9%) demonstrated inhibitory activity, with 33 isolates (52.3%) showing specificity for this Gram-positive bacterium. *C. albicans* growth was inhibited by 39 isolates (11.5%), of which 12 were selective for the yeast alone.

Amid the escalating global crisis of antimicrobial resistance, the discovery of new bioactive compounds is critical. The selective activity observed against *S. aureus*, Gram-negative bacteria (*E. coli* and *P. aeruginosa*), and *C. albicans* highlights the biomedical potential of *Actinomycetota* isolates from the Caatinga biome. These pathogens are listed among high-priority antibiotic-resistant organisms by the WHO, reflecting their widespread resistance and the urgent need for novel therapeutics [21]. Emerging fungal threats, including *Candida auris* and other multidrug-resistant species, further underscore the scarcity of effective antifungal options [22].

Overall, the high prevalence of cultivable, bioactive *Actinomycetota* highlights the Caatinga as a promising, yet underexplored, reservoir of microorganisms capable of producing novel antimicrobial compounds. These findings are in agreement with studies in other extreme habitats, where *Actinomycetota* have consistently yielded diverse metabolites with antibacterial, antifungal, and other bioactive properties [16,17]. While our findings highlight promising antimicrobial activities, it is important to acknowledge that translating crude extracts into clinically useful compounds is a complex and lengthy process, involving isolation, structural characterization, optimization, and rigorous preclinical and clinical evaluation.

### 3.3. Anti-Tubercular Activity of Actinomycetota Isolates from Caatinga Biome

The discovery of streptomycin as the first effective antibiotic against tuberculosis (TB) from *Actinomycetota* initiated extensive exploration of these filamentous microorganisms, leading to a wealth of bioactive compounds [23]. However, the discovery of novel natural products has declined, with roughly half of current antimicrobials originating from the “Golden Age” of the 1950s–1960s. Meanwhile, the emergence of drug-resistant *M. tuberculosis* strains remains a pressing global health challenge, highlighting the need to explore underinvestigated microbial sources. In this study, nineteen *Actinomycetota* isolates (19/340, ~6%) produced metabolites capable of inhibiting *M. tuberculosis* H37Ra (Figure 1C), demonstrating that these isolates represent a promising reservoir for anti-tubercular compounds and reinforcing the value of bioprospecting in extreme or understudied habitats. In addition, we observed that six isolates inhibited the growth of *M. smegmatis* mc^2^155 (6/340, ~1.8%). Notably, all extracts active against *M. tuberculosis* also inhibited *M. smegmatis* (Table 1). *M. smegmatis* is widely used as a surrogate model for *M. tuberculosis* due to its faster growth rate, non-pathogenic nature, and genetic similarity to pathogenic mycobacteria, making it a reliable tool for preliminary screening of anti-tubercular compounds, including those with activity against drug-resistant strains [24].

The antimicrobial screening of Caatinga *Actinomycetota* extracts revealed a selective inhibitory profile against *M. tuberculosis* H37Ra. While some isolates exhibited broad-spectrum activity, inhibiting both Gram-positive and Gram-negative bacteria, the majority of active isolates (14/19, ~74%) showed specificity towards *M. tuberculosis*, as indicated by the dark-shaded cells in Table 1. This selective activity suggests that these *Actinomycetota* produce metabolites with targeted antimycobacterial properties, highlighting their potential as sources of novel compounds for TB drug discovery, particularly for combating multidrug-resistant TB.

**Table 1:** Identification, antimicrobial activity, and GNPS dereplication of *Actinomycetota* isolates from the Caatinga biome.

*Actinomycetota* Isolate ID	Biotyper Identification, Score and Best *Actinomycetota* Matched Pattern	Gram (−)		Gram (+)	Yeast	Mycobacteria		GNPS Dereplication
		*EC*	*PA*	*SA*	*CA*	*Mtb*	*Msmeg*	
I001	NIP */<1.69/*S. violaceoruber*				-	-	-	-
I007	*S. nogalater*/2.24/*S. nogalater*/				-	-	-	-
I021	NIP/<1.69/*S. chartreusis*					-	-	-
I022	NIP/<1.69/*Streptomyces* sp. HKI D58 HKJ					-	-	-
I033	NA					-	-	-
I052	NIP/<1.69/*S. lavendulae*	-	-	-	-		-	-
I068	NIP/<1.69/*S. phaeochromogenes*	-	-	-	-			Actinomycins
I070	NIP/<1.69/*S. nogalater*	-	-	-	-			Actinomycins
I072	*Streptomyces sp.*/1.70/*S. nogalater*					*-*	-	Rabelomycin
I082	NA	-	-	-	-		-	-
I087	*S. nogalater*/2.32/*S. nogalater*					*-*	-	-
I136	NIP/<1.69/*S. violaceoruber*				-			-
I139	NIP/<1.69/*S. violaceoruber*				*-*		-	-
I144	*Streptomyces sp.*/1.76/*S. nogalater*	-	-	-	-		-	-
I152	NIP/<1.69/*S. nogalater*	-	-	-	-		-	-
I159	NA					-	-	-
I184	NIP/<1.69/*S. hirsutus*	-	-	-	-		-	Actinomycins
I185	NIP/<1.69/*S. chartreusis*	-	-	-	-		-	Actinomycins
I217	NIP/<1.69/*S. badius*	-	-	-	-		-	-
I227	NIP/<1.69/*S. chartreusis*	-	-	-	-		-	Actinomycins
I243	NIP/<1.69/*S. badius*				*-*	*-*	-	-
I248	NIP/<1.69/*S. violaceoruber*				*-*		-	-
I251	NIP/<1.69/*S. violaceoruber*	-	-	-	-		-	Cyclomarin A
I271	*Streptomyces sp.*/1.74/*S. violaceoruber*					-	-	-
I299	NIP/<1.69/*S. violaceoruber*	-	-	-	-		-	Cyclomarin A
I300	*Streptomyces sp.*/1.86/*S. violaceoruber*				*-*	*-*	*-*	-
I301	NA				*-*	*-*	*-*	-
I304	*Streptomyces sp.*/1.86/*S. nogalater*					*-*	*-*	-
I307	NIP/<1.69/*S. chartreusis*	-	*-*	*-*	*-*			Actinomycins
I310	NIP/<1.69/*S. hirsutus*	-	-	-	-			Actinomycins
I311	*S. nogalater*/2.02/*S. nogalater*				-	-	-	-
I315	*Streptomyces sp.*/1.94/*S. violaceoruber*				-	-	-	-
I327	*S. violaceoruber*/2.06/*S. violaceoruber*						-	Rabelomycin
I339	NIP/<1.69/*S. chartreusis*							Actinomycins

* NIP: No identification possible, according to the standard Biotyper Bruker interpretative criteria. Abbreviations: *E. coli* (*EC*), *P. aeruginosa* (*PA*), *S. aureus* (*SA*), *C. albicans* (*CA*), *M. tuberculosis* (*Mtb*), *M. smegmatis* (*Msmeg*). The colored area represents growth inhibition. “-”, no growth inhibition. NA, not available.

### 3.4. MALDI-TOF MS-Based Characterization of Actinomycetota Isolates

*Actinomycetota* isolates exhibiting broad-spectrum activity against Gram-positive and Gram-negative bacteria (*n* = 19), as well as those demonstrating inhibitory activity against *M. tuberculosis* (*n* = 19), were characterized by Gram staining. Microscopically, the highly filamentous pseudo-mycelium was stable and unfragmented, consistent with characteristics of probable *Streptomyces* species, as described in Bergey’s Manual of Systematic Bacteriology (Supplementary Figure S3A,B) [25]. In addition to classical morphological analyses, the isolates were characterized using mass spectra acquired with a MALDI-TOF mass spectrometer.

Three isolates were identified as *Streptomyces nogalater* (I007, I087 and I311) and one as *Streptomyces violaceoruber* (I327) with a score above 2.0, supporting secure genus-level and probable species-level identification (Table 1). All three *Streptomyces nogalater* isolates exhibited a comparable inhibition profile, active against Gram-negative and Gram-positive bacteria but inactive against *M. tuberculosis* and *M. smegmatis*. This concordance reinforces that the isolates are closely related, likely representing the same species, and suggests that they share a similar repertoire of bioactive metabolites. Six isolates (I072, I144, I271, I300, I304 and I315) yielded scores between 1700 and 1999, which corresponds to probable genus-level identification, and were classified as *Streptomyces* spp. (Table 1). The assignment at the genus level reflects the remarkable taxonomic diversity within *Streptomyces*, a genus known for harboring numerous closely related species with overlapping phenotypic and biochemical traits. This finding suggests that the isolates may represent putatively distinct species within the genus, potentially including strains with unexplored metabolic capabilities and the ability to produce novel bioactive compounds. However, the method was unable to determine the taxonomic identity of the remaining isolates (~70%), with scores below 1.699. Despite the low scores obtained, the software indicated the best-matched patterns with species of the genus *Streptomyces*, suggesting a likely affiliation even with limited or low-confidence data (Table 1).

MALDI-TOF MS has been widely adopted in clinical microbiology laboratories and is recognized as a rapid, reliable, and cost-effective method for bacterial species identification [26]. Moreover, numerous studies have validated the performance of MALDI-TOF MS for the identification of environmental bacteria through comparison with molecular techniques and have concluded that this technique is useful and applicable to environmental bacteria [27]. However, in this study, the MALDI-TOF MS-based methodology proved largely unsuccessful for the identification of the majority of isolates. This limitation is primarily attributable to the low number of reference *Actinomycetota* spectra in database libraries. An equally compelling explanation is that *Actinomycetota* from the Caatinga biome are highly divergent from previously characterized strains, likely as a direct adaptive response to extreme cyclical stresses. This divergence presumably drives the evolution of specialized biosynthetic pathways essential for survival and competition in this oligotrophic environment.

Therefore, our results highlight a critical gap in current microbiological resources. The low MALDI-TOF MS identification scores obtained here underscore the limitation of relying solely on this method for taxonomic identification of microbes from underexplored environments. Consequently, this finding highlights the need for genomic analyses (e.g., 16S rRNA or whole-genome sequencing) to accurately determine their phylogenetic placement and confirm the potential novelty suggested by our data. In parallel, multi-institutional initiatives aimed at creating and sharing specialized mass spectrometry datasets for *Actinomycetota* from extreme biomes are crucial. Such efforts will greatly enhance the high-throughput characterization of new strains and foster taxonomically guided discovery of novel natural products from these promising sources [28].

### 3.5. Metabolomic Fingerprint of Antibiotic-Producing Actinomycetota from Caatinga

To investigate whether the observed bioactivities could be attributed to known metabolites present in the extracts, or instead suggest the presence of novel natural products, we performed a dereplication analysis of 34 active extracts (Table 1). Over the last decade, molecular networking via the GNPS platform has become a routine and indispensable tool for the exploratory analysis of large extract libraries, fundamentally changing the workflow of natural product discovery [29,30]. For this purpose, the GNPS molecular networking platform was employed, enabling automated detection of MS/MS-based structural relatedness among features. The resulting molecular networks grouped spectra into families, linking unknown spectra to reference spectra of known compounds based on cosine similarity [11]. Currently, often allied with other computational metabolomics methods, this strategy has enabled the discovery of numerous novel bioactive metabolite classes from diverse sources [11,30]. This approach was applied both for dereplication and for the construction of molecular networks, allowing the identification of spectral families associated with known secondary metabolites.

**Figure 2:**
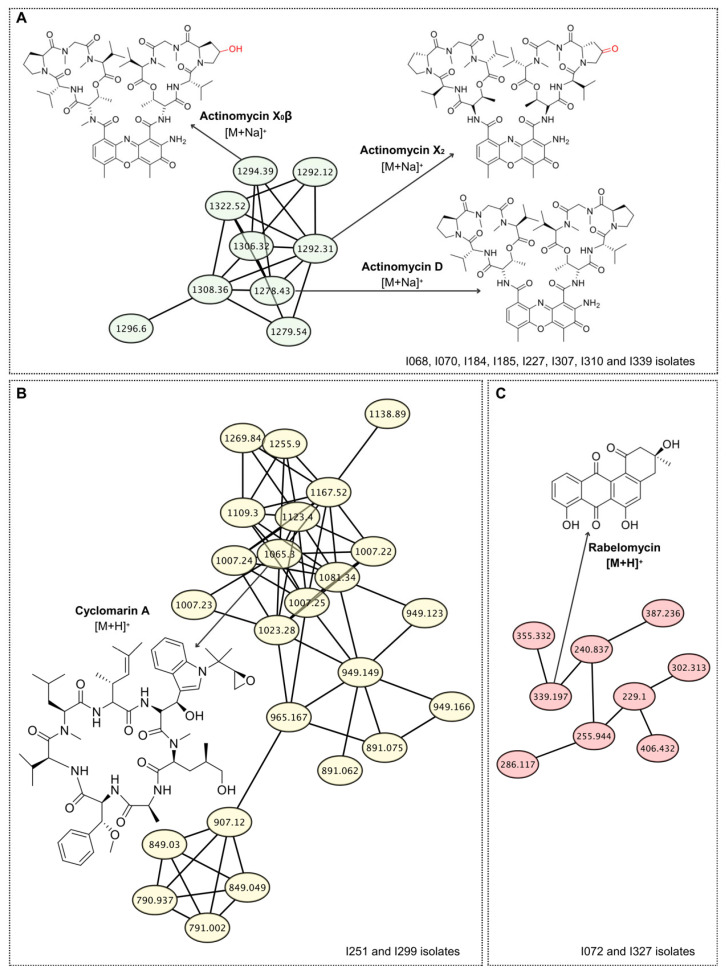
**Molecular networking unveils the specialized metabolome of *Actinomycetota* strains, highlighting known and variant antibiotics**. LC-MS/MS data from *Actinomycetota* extracts were processed using GNPS to generate molecular families based on MS/MS spectral similarity. (**A**) A cluster annotated to the actinomycin family, with nodes corresponding to actinomycin D, X_0_β, and X_2_, identified by spectral matching (cosine score > 0.7). (**B**) A distinct molecular family led to the identification of the antimycobacterial cyclic peptide cyclomarin A. (**C**) Annotation of a cluster as the angucycline-class antibiotic rabelomycin, suggesting the potential for related derivatives. All annotations were confirmed by comparison to reference MS/MS spectra from public spectral libraries.

Using this approach, eight *Actinomycetota* isolates (I068, I070, I184, I185, I227, I307, I310 and I339) were found to produce one or more members of the actinomycin family (Table 1). Actinomycins are chromopeptide antibiotics belonging to the depsipeptide class, characterized by a phenoxazinone chromophore linked to a cyclic peptide lactone [31]. They represent a well-known group of secondary metabolites widely produced by diverse *Streptomyces* species and are recognized for their potent antibacterial and antitumor properties [32]. Analysis of the mass spectra of the eight isolates confirmed the presence of actinomycin D (*m*/*z* 1255.6 [M+H]^+^), actinomycin X_2_ (*m*/*z* 1269.6 [M+H]^+^) and actinomycin X_0_β (*m*/*z* 1271.6 [M+H]^+^) in all samples annotated by GNPS, demonstrating consistency of the dereplication data. Importantly, inspection of the GNPS molecular network revealed six additional clusters of related molecules beyond the three known actinomycins, strongly suggesting that the isolates also produce actinomycin D analogues (Figure 2A). This finding not only underscores the metabolic richness of the strains but also highlights their potential as a valuable source of structurally diverse and bioactive natural products with relevance for antibiotic discovery.

Although actinomycins are well known for their antibacterial activity against Gram-positive bacteria, such as *S. aureus*, only isolate I339 displayed inhibitory activity in the agar plug test (Table 1). This discrepancy may be related to the amount and specific type of actinomycin produced by different *Streptomyces* species. For example, actinomycin D exhibits approximately two-fold-lower potency against *S. aureus* compared to actinomycin X_2_, highlighting that structural variants can significantly influence antibacterial efficacy [33].

The eight *Actinomycetota* isolates producing actinomycins were active against *M. tuberculosis*, whereas only five of them exhibited activity against *M. smegmatis*. In this context, the identification of actinomycin-producing *Streptomyces* isolates presents a promising avenue for the discovery of new anti-TB agents. Nonetheless, to translate these findings into viable treatments, it is imperative to focus on the development of actinomycin analogs with reduced toxicity, thereby expanding their clinical applicability and contributing to the global effort against drug-resistant TB.

Extracts from isolates I251 and I299 displayed spectra consistent with cyclomarin A, and molecular networking analysis further grouped additional related spectra (Figure 2B), suggesting that these isolates may produce not only cyclomarin A but also novel structural analogues of this molecule. Moreover, both extracts showed selective inhibition of *M. tuberculosis* growth, reinforcing cyclomarin-type metabolites as highly promising scaffolds for the development of next-generation anti-tubercular drugs. Cyclomarin A is a cyclic heptapeptide originally isolated from a marine *Streptomyces* species and belongs to the family of nonribosomal peptides characterized by unusual amino acid residues and structural complexity [34]. It has attracted considerable attention due to its potent and selective antimycobacterial activity, particularly against *M. tuberculosis* [35]. Mechanistic studies revealed that cyclomarin A targets the essential protease ClpC1, a component of the caseinolytic protease complex, leading to dysregulation of protein homeostasis and bacterial death [36]. To our knowledge, this is the first report of cyclomarin A detection in terrestrial *Actinomycetota*, highlighting the Caatinga biome as a promising reservoir of novel bioactive scaffolds for anti-tubercular drug discovery.

Rabelomycin, a member of the aromatic polyketide angucycline family, is characterized by its benz[a]anthracene tetracyclic skeleton. It is generally considered an intermediate or shunt product in the biosynthesis of more complex angucyclines, such as urdamycins and landomycins [37,38]. Although not widely recognized for potent antibacterial activity on its own, rabelomycin exhibits significant cytotoxic and antiproliferative properties, making it a compound of potential interest in cancer research [39]. Its frequent detection in metabolomic studies also establishes it as a reliable chemotaxonomic marker for angucycline-producing *Actinomycetota* [38]. In this study, two isolates, I072 and I327, displayed mass spectra consistent with rabelomycin, as well as other related angucycline compounds, as evidenced by the GNPS molecular network (Figure 2C). This finding corroborates the conserved presence of angucycline-type metabolites in *Streptomyces* isolates and highlights the genus’s potential as a reservoir for structurally diverse bioactive molecules. Both isolates, I072 and I327, exhibited a broad-spectrum antimicrobial profile, inhibiting the growth of Gram-positive and Gram-negative bacteria, as well as *C. albicans*. This pattern aligns with the known activity of angucyclines, such as rabelomycin, which have demonstrated antibacterial effects against Gram-positive bacteria [40]. While rabelomycin’s antifungal activity is less documented, some studies have reported antifungal properties in related compounds [41].

**Figure 3:**
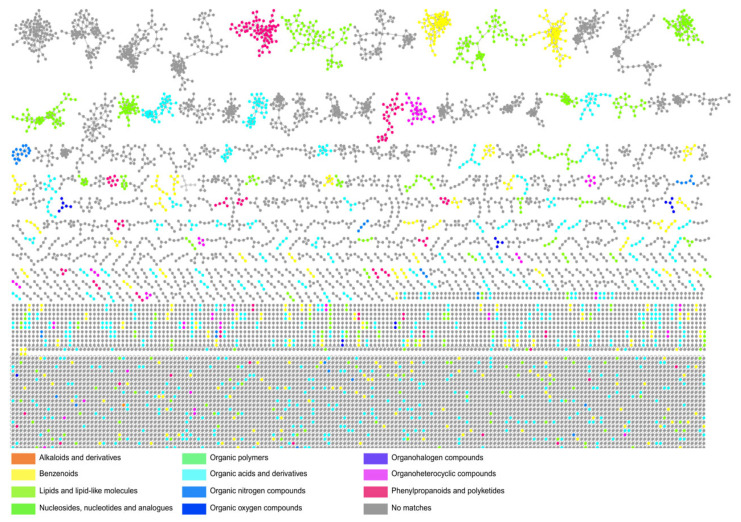
**Molecular networking analysis and chemical annotation of specialized metabolites from *Actinomycetota* extracts.** Molecular networks were generated from LC-MS/MS data processed using GNPS. Nodes represent molecular families (clusters) of MS/MS spectra grouped by structural similarity. Chemical annotation was performed using the MolNetEnhancer workflow, which integrates: (i) spectral library matching, (ii) in silico molecular class prediction via CANOPUS (Class Assignment and Ontology Prediction Using Mass Spectrometry), and (iii) chemical substructure annotation via NAP (Network Annotation Propagation). Node colors represent the major chemical classes identified, highlighting a diverse metabolome that includes several clusters of specialized metabolites putatively annotated.

Notably, the majority of molecular families detected in the GNPS-generated network could not be assigned to known compounds (Figure 3). This pattern closely mirrors findings from Ribeiro et al. (2020), where, after dereplication of 26 *Actinomycetota* extracts exhibiting antimicrobial or cytotoxic activity, 19 showed no database matches; further molecular networking revealed 11 clusters unique to single extracts, many of which were considered prime candidates for novel natural products [42]. Likewise, in our study, most of the detected molecular clusters could not be annotated based on existing spectral libraries (dereplication-resistant compounds), underscoring the presence of chemically uncharted molecular families and reinforcing the potential of the Caatinga biome as a repository of potential and strong targets for the future isolation and elucidation of new natural products.

## 4. Conclusions

Our findings firmly establish the Brazilian Caatinga biome as an untapped reservoir of bioactive *Actinomycetota*. The high recovery rate of isolates exhibiting potent and selective activity against WHO priority pathogens, including *M. tuberculosis*, underscores the strategic value of bioprospecting in extreme environments. While taxonomic characterization revealed a likely dominance of *Streptomyces*, the limited database matches suggest a wealth of novel diversity awaiting description.

Critically, our integrated approach, which combines classical microbiology with modern metabolomics, proved highly effective. GNPS molecular networking not only suggested the production of potent known antibiotics like actinomycins and the anti-TB cyclomarin A but, more importantly, revealed a vast uncharted chemical landscape dominated by molecular families with no match to existing databases.

This work provides a robust foundation for future drug discovery efforts. The prioritized isolate collection represents a high-value starting point for the isolation and structural elucidation of novel anti-infective agents, directly contributing to the global arsenal against antimicrobial resistance.

## Data Availability

Data is contained within the article or Supplementary Material. The original contributions presented in this study are included in the article/Supplementary Material. Further inquiries can be directed to the corresponding author(s).

## Supplementary Materials

The following supporting information can be downloaded at https://www.mdpi.com/article/10.3390/life15101494/s1, Figure S1: Study area, climatic features, and *Actinomycetota* isolation from Caatinga soils.; Figure S2: Number of *Actinomycetota* colonies recovered from each soil sample (#1–#6).; Figure S3: *Actinomycetota* isolates.
