# Supplementary material for: The Brazilian Caatinga Biome as a Hotspot for the Isolation of Antibiotic-Producing Actinomycetota
Source: Life (Basel). 2025 Sep 23;15(10):1494. doi: 10.3390/life15101494 (PMC12565491; doi:10.3390/life15101494)
Supplement: Supplementary file 1 [file life-15-01494-s001.zip › life-3874351-supplementary.pdf]

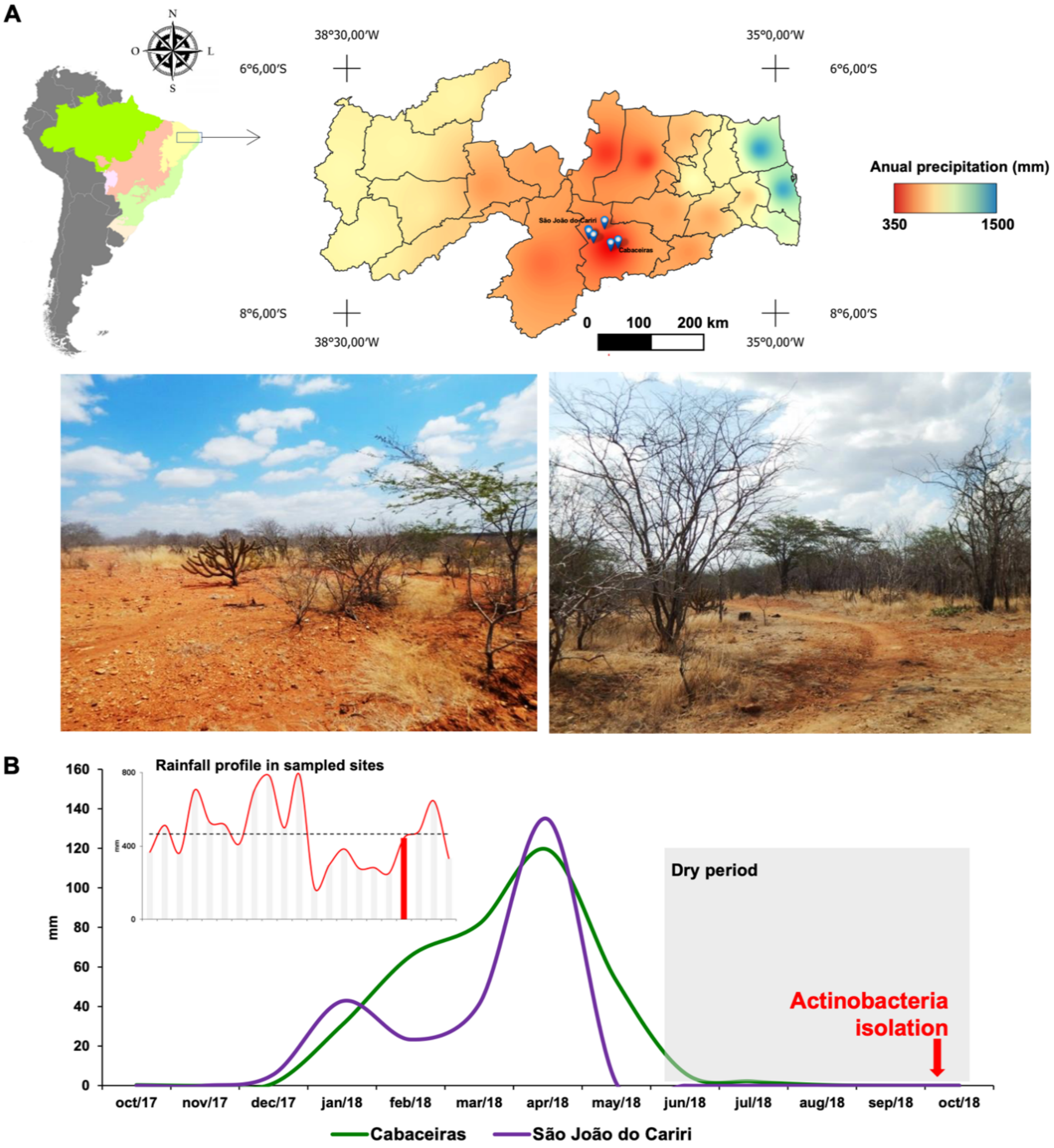

Supplementary Figure S1. Study area, climatic features, and Actinomycetota isolation from Caatinga soils. (A) Geographic location of the study area. On the left, Brazilian biomes are shown with the Caatinga biome highlighted in yellow. The inset map displays annual precipitation in Paraíba State and the six soil collection sites (#1-#6). Representative landscapes of the Caatinga morphoclimatic domain are shown below (sites #3 and #5). (B) Rainfall patterns of the sampled municipalities between October 2017 and October 2018. Inset: mean annual rainfall profile for the region (2001-2021), with accumulated precipitation for 2018 indicated in red and the historical mean represented by a dashed line. The dry period during which soil sampling was performed is highlighted.

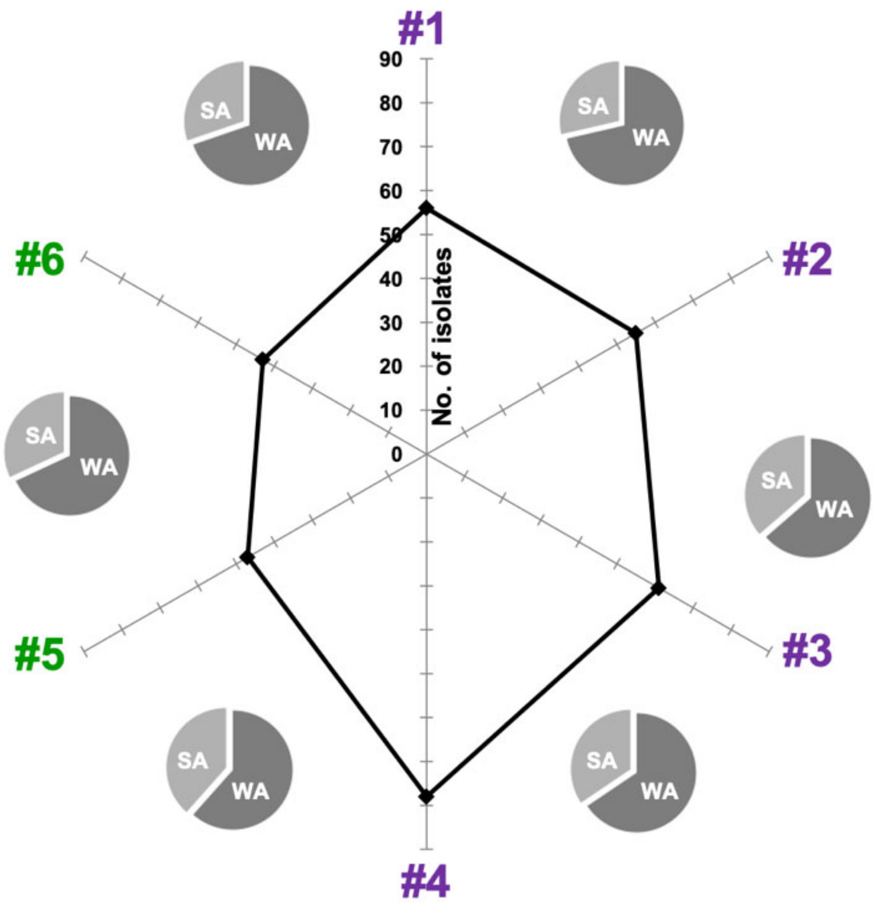

Supplementary Figure S2. Number of Actinomycetota colonies recovered from each soil sample (#1-#6). Pie charts indicate the relative frequency (%) of isolates obtained from different culture media (WA: Water-agar; SA: Soil-agar).

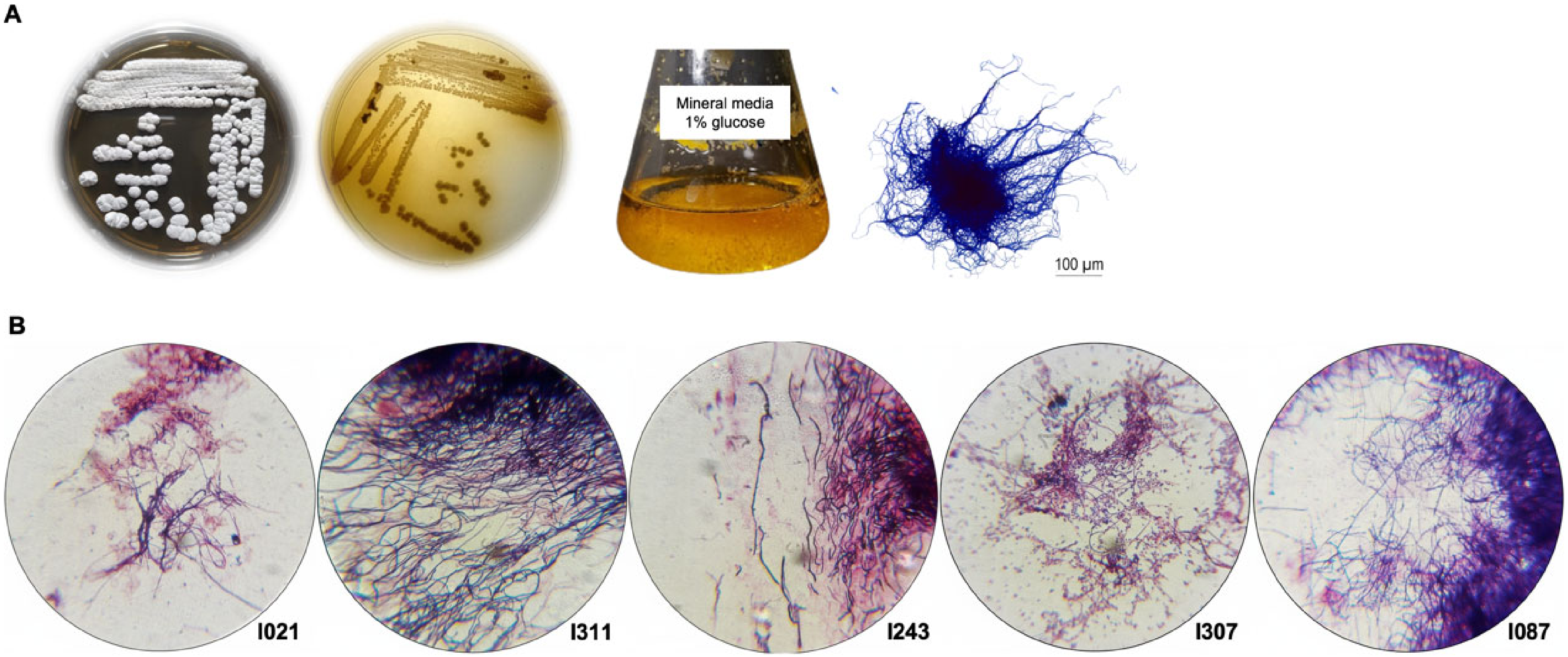

Supplementary Figure S3. Actinomycetota isolates. (A) Representative Actinomycetota isolate (I339) cultivated in yeast malt extract medium (ISP2, left Petri dish), mineral medium M9 (right Petri dish), and M9 liquid culture. From the liquid culture, pseudomycelia stained with methylene blue are shown. (B) Gram staining of Actinomycetota isolates. Photomicrographs illustrate key morphological traits: fragmentation of substrate pseudohyphae (arrows) and spores (arrowhead).
